# Supplementary figures and images for: Effects of auxin derivatives on phenotypic plasticity and stress tolerance in five species of the green alga Desmodesmus (Chlorophyceae, Chlorophyta)
Source: PeerJ. 2020 Mar 9;8:e8623. doi: 10.7717/peerj.8623 (PMC7067201; doi:10.7717/peerj.8623)

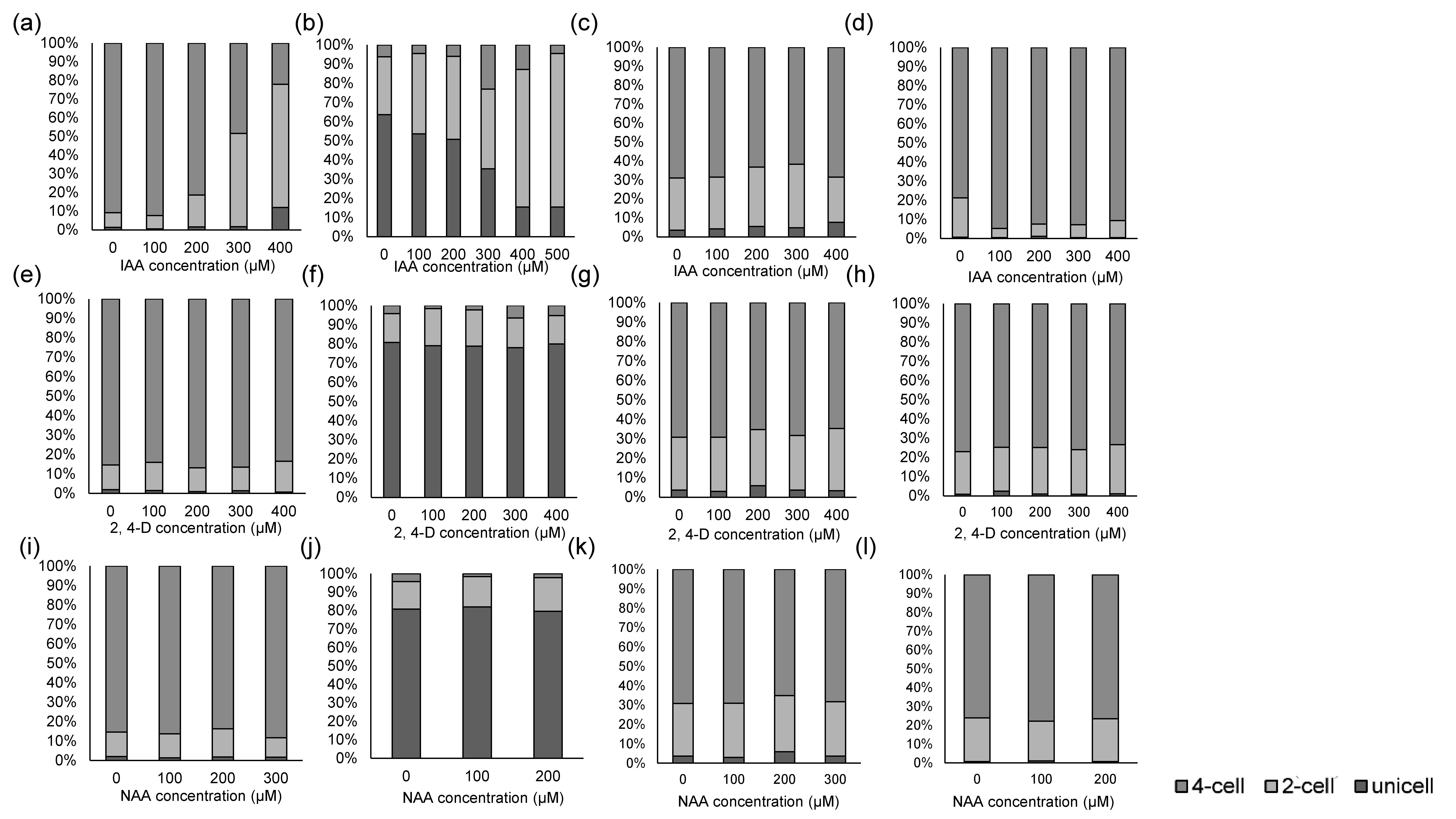

Supplement: Supplemental Information 1 — Data are presented as means (n = 3) for each group, and morphotype percentages and cell types were based on 200 cell counts in each repeat. Means with the same letter are not significantly different from each other according to the results of a one-way analysis of variance and least significant difference post hoc test. a, d, g D. armatus JYCA037. b, e, h D. armatus JYCA041. c, f, i D. armatus JYCA045. [file peerj-08-8623-s001.png]

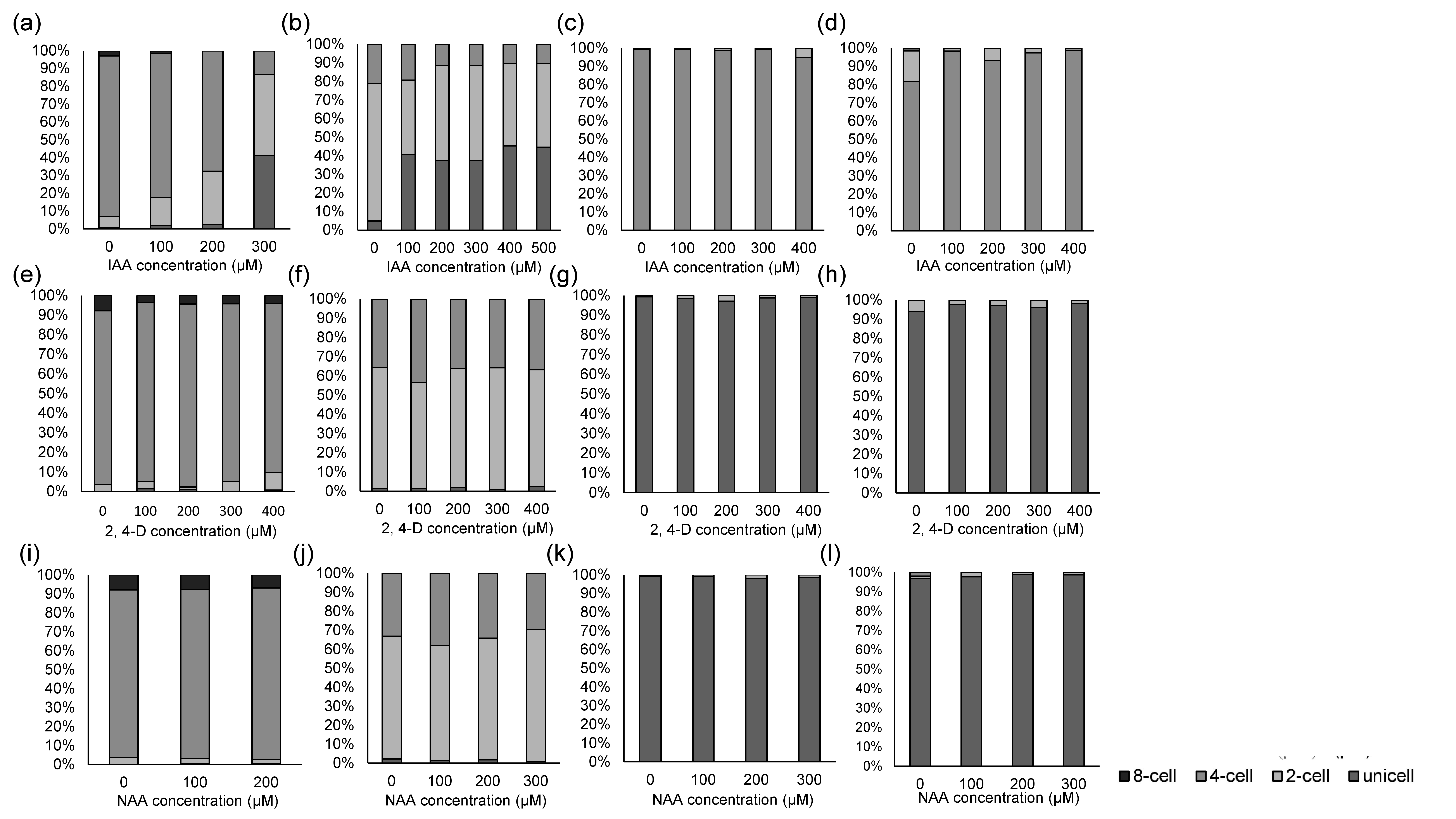

Supplement: Supplemental Information 2 — Data are presented as means (n = 3) for each group, and morphotype percentages and cell types were based on 200 cell counts in each repeat. Means with the same letter are not significantly different from each other according to a one-way analysis of variance and least significant difference post hoc test. a, f, kD. armatus JYCA039. b, g, l D. communis JYCA040. c, h, m D. opoliensis JYCA043. d, i, n D. communis JYCA044. e, j, oD. intermedius strain JYCA042. [file peerj-08-8623-s002.png]

Fig. 3a


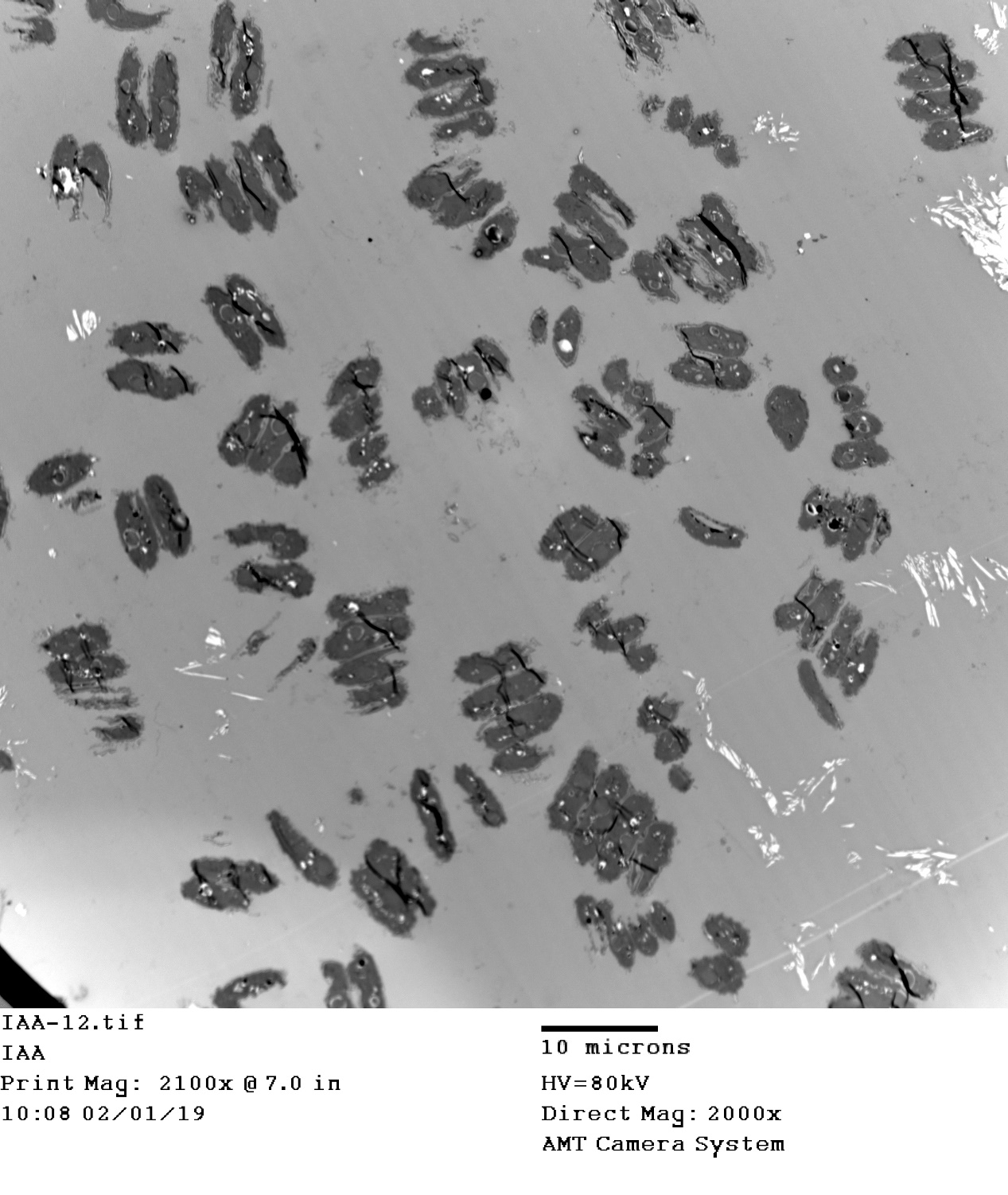


Fig. 3b


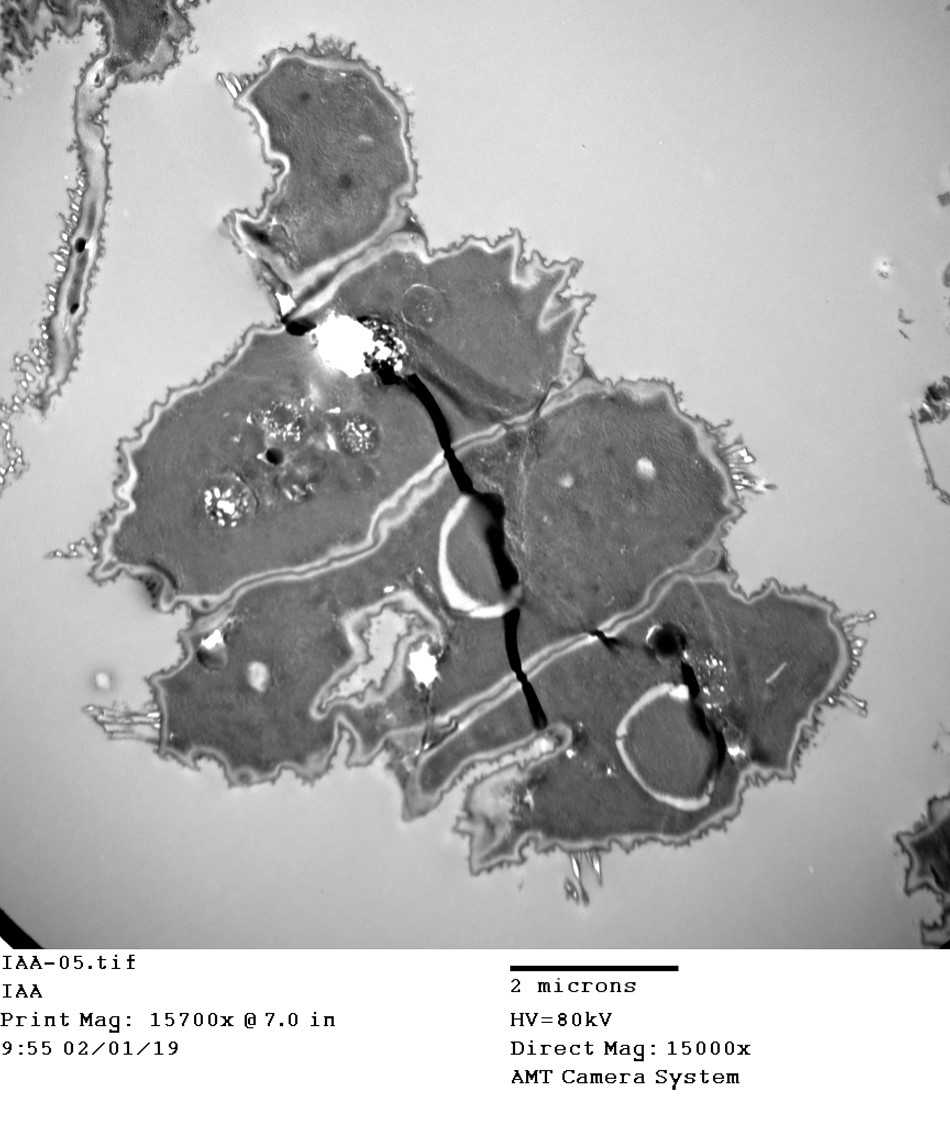


Fig. 3c


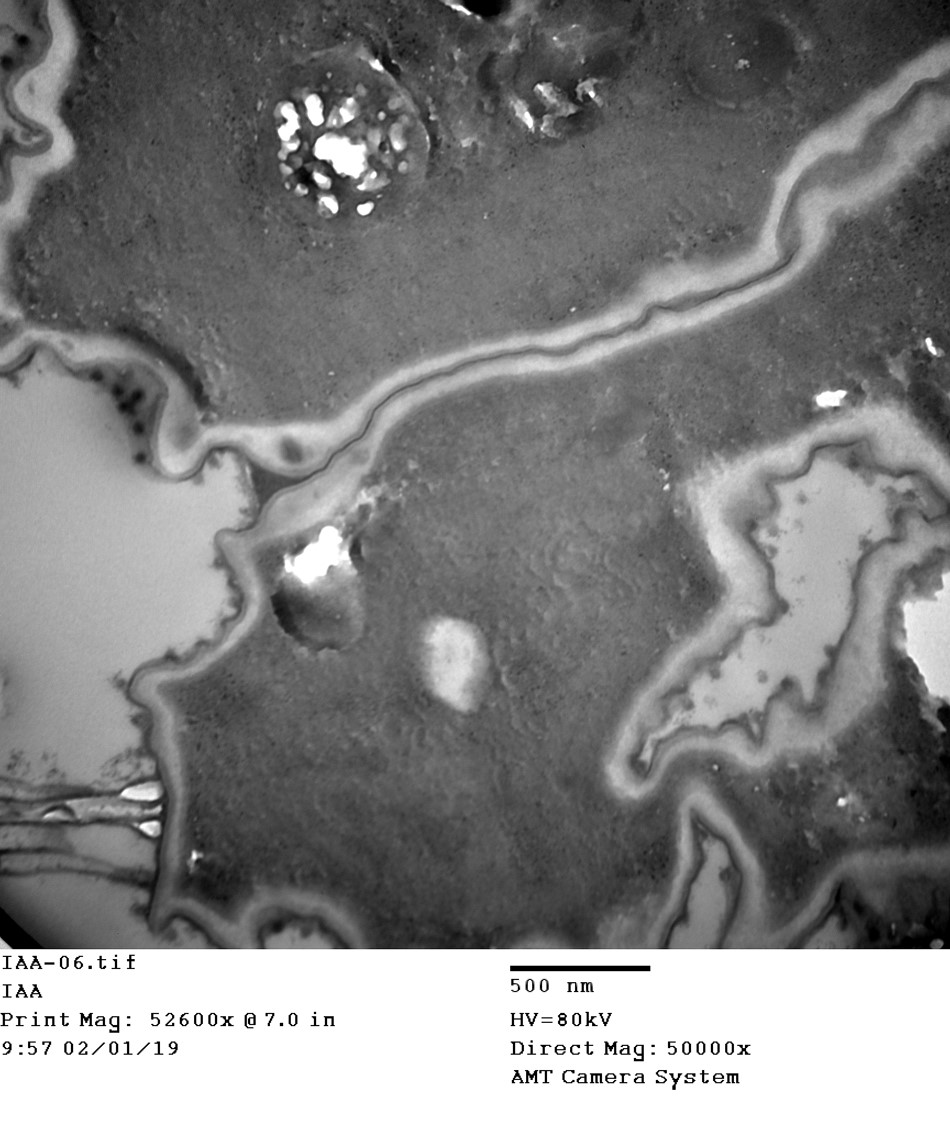


Fig. 3d


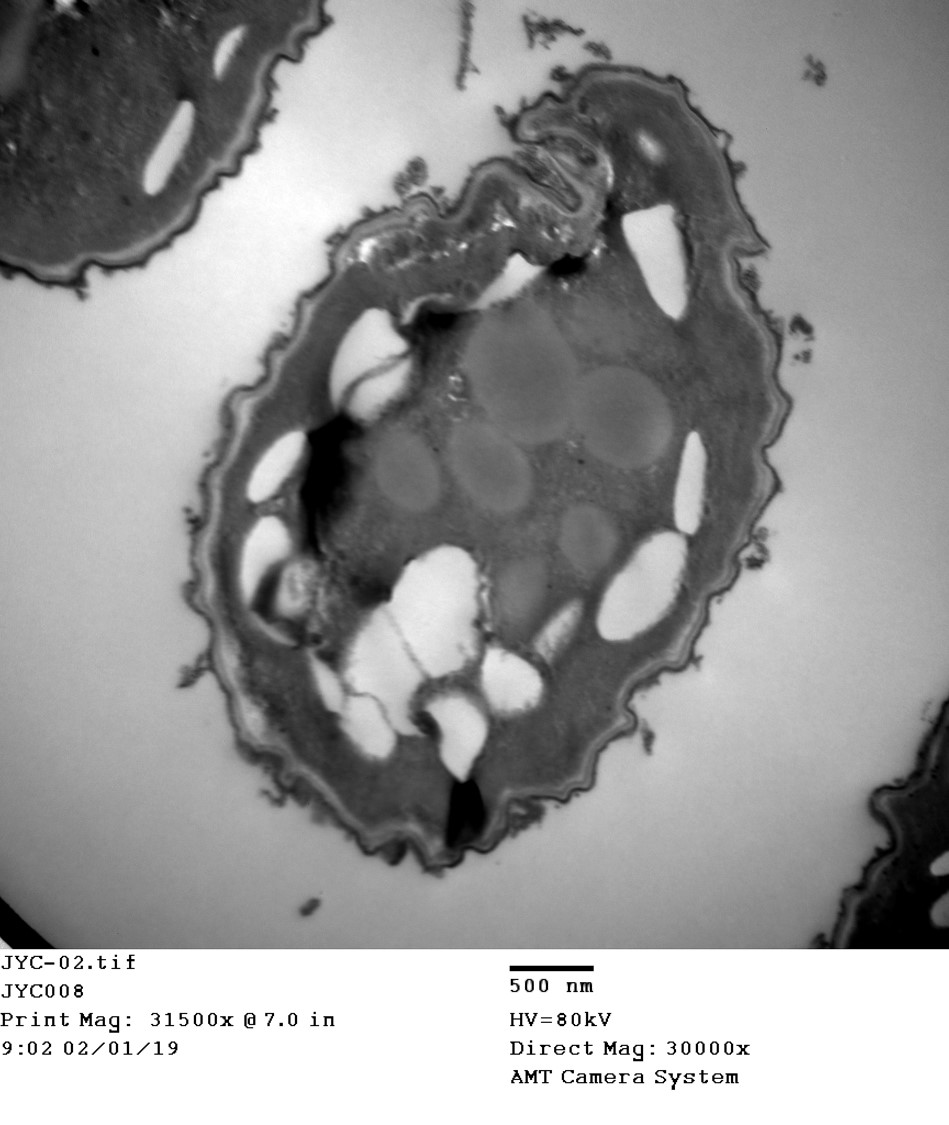

Supplement: Supplemental Information 6 [file peerj-08-8623-s006.docx]
