# Supplementary material for: Effects of auxin derivatives on phenotypic plasticity and stress tolerance in five species of the green alga Desmodesmus (Chlorophyceae, Chlorophyta)
Source: PeerJ. 2020 Mar 9;8:e8623. doi: 10.7717/peerj.8623 (PMC7067201; doi:10.7717/peerj.8623)
Supplement: Supplemental Information 3 — Names listed in this study were checked against according to algaebase (https://www.algaebase.org/). [file peerj-08-8623-s003.docx]

Supplementary Table 1、Algal strains used in this study with their accession numbers of the SSU and ITS regions and their ecological sources. Names listed in this study were checked against according to algaebase (https://www.algaebase.org/).

| **strains** | **closest species (GenBank accession no.) (18S)** | **nucleotide substitutions/ total nt** | **closest species (GenBank accession no.) (ITS)** | **nucleotide substitutions/ total nt** | **species** | **accession no. (18S/ITS)** | | **ecological sources** |
| --- | --- | --- | --- | --- | --- | --- | --- | --- |
| JYCA008 | *Desmodesmus komarekii* (AB818541) | 790/790 | *Desmodesmus komarekii* (DQ417562) | 594/599 | *Desmodesmus komarekii* | MN817683 | MN759324 | bark of Alstonia scholaris, Changhua County, Taiwan |
| JYCA037 | *Scenedesmus armatus* ( KR082490) | 753/753 | *Scenedesmus armatus* (MF346374) | 615/615 | [*Desmodesmus armatus*](http://algaebase.org/search/species/detail/?species_id=i1f4f583d11aa162d&sk=0&from=results) | MN817678 | MN759319 | artificial pond, Changhua County, Taiwan |
| JYCA038 | *Desmodesmus pannonicus* (MK541797) | 747/748 | *Desmodesmus pannonicus* (FR865712) | 635/638 | *Desmodesmus pannonicus* | MN817679 | MN759320 | artificial pond, Changhua County, Taiwan |
| JYCA039 | *Desmodesmus armatus* (MK541735) | 794/795 | *Desmodesmus armatus* (AB917113) | 608/609 | *Desmodesmus armatus* | MN817680 | MN759321 | artificial pond, Changhua County, Taiwan |
| JYCA040 | *Desmodesmus communis* (LC472545) | 697/698 | *Desmodesmus communis* (KP726233) | 600/604 | *Desmodesmus communis* | MN817681 | MN759322 | artificial pond, Changhua County, Taiwan |
| JYCA041 | *Desmodesmus armatus* (MK541798) | 691/692 | *Scenedesmus armatus* (JQ910904) | 620/624 | *Desmodesmus armatus* | MN817682 | MN759323 | artificial pond, Changhua County, Taiwan |
| JYCA042 | *Desmodesmus intermedius* (MK764918) | 969/980 | *Desmodesmus spinosus*(KT778091) | 597/632 | *Desmodesmus intermedius* | MN817674 | MN759315 | artificial pond, Changhua County, Taiwan |
| JYCA043 | *Desmodesmus insignis* (KU175228) | 639/720 | *Desmodesmus opoliensis* (AB917108) | 621/624 | *Desmodesmus opoliensis* | MN817675 | MN759316 | artificial pond, Changhua County, Taiwan |
| JYCA044 | *Desmodesmus communis* (LC472545) | 727/728 | *Desmodesmus spinosus (*KT778091) | 596/631 | *Desmodesmus communis* | MN817676 | MN759317 | artificial pond, Changhua County, Taiwan |
| JYCA045 | *Desmodesmus armatus* (MK541735) | 856/857 | *Desmodesmus armatus* ( AB917113) | 625/627 | *Desmodesmus armatus* | MN817677 | MN759318 | artificial pond, Changhua County, Taiwan |
