# Supplementary material for: Effects of auxin derivatives on phenotypic plasticity and stress tolerance in five species of the green alga Desmodesmus (Chlorophyceae, Chlorophyta)
Source: PeerJ. 2020 Mar 9;8:e8623. doi: 10.7717/peerj.8623 (PMC7067201; doi:10.7717/peerj.8623)
Supplement: Supplemental Information 4 [file peerj-08-8623-s004.docx]

**This page documents our supplementary material for the PeerJ submission:**

*Isolation and Culture of Microalgae:*

The algal strains used here were isolated from natural water bodies in Central Taiwan. Water samples with visible microalgal population were centrifuged at 3000 ×*g* for 10 minutes at room temperature to concentrate the cells and spread onto CA agar plates (with 0.8% w/v agar). For isolating an axenic single colony from field water samples, the streak plate method was used. The algae were cultured in CA medium, consisting of 2 mg/L Ca(NO_3_)_2_.4H_2_O, 10 mg/L KNO_3_, 5 mg/L NH_4_NO_3_, 3 mg/L β–Na_2_glycerophosphate.5H_2_O, 2 mg/L MgSO_4_.7H_2_O, 0.01 μg/L vitamin B12, 0.01 μg/L biotin, 1 μg/L thiamine HCl, and 0.1 mL/L PIV metals (1000 mg/L Na_2_EDTA.2H_2_O, 196 mg/L FeCl_3_.6H_2_O, 36 mg/L MnCl_2_.4H_2_O, 10.4 mg/L ZnCl_2_, 4 mg/L of CoCl_2_.6H_2_O, and 2.5 mg/L of Na_2_MoO_4_.2H_2_O), 0.1 mL/L Fe (as EDTA; 1:1 molar; 702 mg/L Fe(NH_4_)_2_(SO_4_).6H_2_O and 660 mg/L Na_2_EDTA.2H_2_O), and 40 mg/L of HEPES; all compounds were directly mixed, the pH was then adjusted to 7.2 and autoclaved (15 min at 120°C). Isolated algal cells were stored at −80°C in 15%–20% glycerol. For each experiment, the alga was cultured axenically in liquid CA medium at 125 rpm in a tube rotator and grown at 25°C under cool white fluorescent light (approximately 46.30 µmol m^−2^ s^−1^) with a 14:10-h light–dark period. Each algal culture sample was observed for cellular growth rates by measuring the optical density at 680 nm. The regression equation between cell density (y × 10^5^/mL) and OD_685_ (x) was derived as y = 162.1x + 1.3463 (r^2^ = 99.34%) (Qian et al., 2009).
